# The Effectiveness of Spinal, Diaphragmatic, and Specific Stabilization Exercise Manual Therapy and Respiratory-Related Interventions in Patients with Chronic Nonspecific Neck Pain: Systematic Review and Meta-Analysis

**DOI:** 10.3390/diagnostics12071598

**Published:** 2022-06-30

**Authors:** Petros I. Tatsios, Eirini Grammatopoulou, Zacharias Dimitriadis, Maria Papandreou, Eleftherios Paraskevopoulos, Savvas Spanos, Palina Karakasidou, George A. Koumantakis

**Affiliations:** 1 Physiotherapy Department, School of Health & Care Sciences, University of West Attica (UNIWA), 12243 Athens, Greece; igrammat@uniwa.gr (E.G.); mpapand@uniwa.gr (M.P.); eparaskevop@uniwa.gr (E.P.); pkarakasidou@uniwa.gr (P.K.); gkoumantakis@uniwa.gr (G.A.K.); 2 Laboratory of Advanced Physiotherapy, Physiotherapy Department, School of Health & Care Sciences, University of West Attica (UNIWA), 12243 Athens, Greece; 3 Health Assessment & Quality of Life Laboratory, Physiotherapy Department, University of Thessaly, 35100 Lamia, Greece; zdimitriadis@uth.gr; 4 Human Performance & Rehabilitation Laboratory, Physiotherapy Department, University of Thessaly, 35100 Lamia, Greece; sspanos@uth.gr

**Keywords:** chronic neck pain, diaphragm, manual therapy, breathing exercises, respiratory dysfunction, breathing re-education

## Abstract

Patients with nonspecific chronic neck pain (NSCNP) exhibit respiratory dysfunction. This systematic review aimed to analyze randomized controlled trials (RCTs) investigating the effect of spinal and/or diaphragmatic and/or specific stabilization exercise manual therapy and/or respiratory exercises on musculoskeletal and respiratory diagnostic outcomes in patients with NSCNP. A systematic search and selection of RCTs was performed in three scientific databases (Pubmed, Scopus, and Physiotherapy Evidence Database (PEDro)) and one search engine (Google Scholar) from inception to April 2022. Relevant studies published in the English language were extracted, evaluated, and independently rated for methodological quality (PEDro scale). The quality of the evidence was assessed with the GRADE approach. Out of 1089 studies collected in total, 1073 were excluded (i.e., did not meet the inclusion criteria or were duplicates). Sixteen RCTs were finally included, rated on 5.62/10 (PEDro score) on average for methodological quality. Overall, there was sparse evidence that spinal and/or diaphragmatic manual therapy and/or trunk stabilization exercises and/or respiratory exercises significantly improved pain, disability, and respiratory outcomes in patients with NSCNP immediately post-treatment. However, the clinical heterogeneity between studies was significant, and the level of certainty of the evidence was low to very low. More, high-quality RCTs are required, contributing to the holistic diagnostic monitoring and management of patients with NSCNP.

## 1. Introduction

Chronic neck pain (CNP) is reported to be one of the most common musculoskeletal pain syndromes, with an annual prevalence estimated to range between 30% and 50% worldwide [1]. Data derived from the Global Burden of Diseases, Injuries, and Risk Factors Study 2019 highlight that neck pain was in the top five, ranking among musculoskeletal disorders, with 223 million affected people worldwide and 22 million years of life lived with disability [2]. CNP may affect the individual’s physical, social, and psychological well-being, contributing to an increase in costs to society and business. CNP is a significant cause of morbidity and disability in everyday life and at work in many countries, but its primary pathology and pathophysiology are still unclear [1,3]. 

Nonspecific neck pain is defined as pain and discomfort localized between the superior nuchal line and the spinous process of the first thoracic vertebra. The pain may radiate to the scapula region, anterior chest wall, head, or upper limb [4]. Nonspecific chronic neck pain (NSCNP) is defined as neck pain persisting for longer than 12 weeks or after the healing period or recurring neck pain that intermittently affects an individual over a long time [5]. The primary source of the pain could be the zygapophysial joints, the intervertebral disc, or the surrounding soft tissues of the area [4]. 

A comprehensive diagnostic approach must be adopted for patients with NSCNP in order to monitor the effectiveness of rehabilitation interventions. People with NSCNP present with local hyperalgesia, impaired conditioning pain modulation, psychological disruptions (depressive symptoms, pain catastrophizing), low quality of life, reduced active range of motion, and alterations in the timing and activation of the cervical muscles [6]. Furthermore, cervical muscles contributing to respiration (sternocleidomastoid, anterior scalene, trapezius) tend to exhibit reduced function due to pain in terms of strength, endurance, motor control, and proprioception [6,7]. Neck pain may cause altered neck muscle sequence of activation (incoordination patterns) and altered neck muscle activation levels (reduced activation of the deep segmental muscles and increased activation of the superficial muscles) [3,7]. In some patients with NSCNP, the above-mentioned changes may lead to a poor breathing pattern, potentially altering pH blood levels, causing smooth respiratory muscle constriction, altered electrolyte balance, decreased tissue oxygenation [8], hypertonia of the accessory muscles, hypomobility of the thoracic cage, shortening of the accessory muscles, and forward head posture, contributing to the pain experience [9]. Respiratory dysfunction presents in the form of changes in the blood chemistry and dysfunction of respiratory muscles, with concomitant decreases in inspiratory strength and associated respiratory outcomes (volumes/capacities, flows), such as maximum voluntary ventilation (MVV), forced vital capacity (FVC), forced expiratory volume in the first second of expiration (FEV1), end tidal CO2 (ETCO2), maximum inspiratory pressure (MIP), and maximum expiratory pressure (MEP) [7,8,9]. However, researchers have previously estimated the effect size of the respiratory dysfunction concerning these respiratory parameters in patients with NSCNP to be moderate [9].

Although patients with NSCNP have conventionally been treated by traditional physical therapy, education, general exercise, or manual therapy (spinal mobilization, manipulation techniques, and specific exercise) [10], the majority of patients reach a plateau and do not completely recover from their symptoms [8]. Based on the current evidence underlying the observed respiratory dysfunction in patients with NSCNP [9], a previous cohort study incorporating breathing management demonstrated improvement in musculoskeletal and respiratory outcomes, with full resolution of symptoms [8]. According to the International Federation of Orthopedic Manipulative Physical Therapists (IFOMPT), manual therapy (MT) is a specialized area of physical therapy for the management of neuromusculoskeletal conditions based on clinical reasoning and using precise treatment approaches, including manual techniques and specific therapeutic exercises, while, in parallel, considering the psychosocial state of each patient [11]. 

Several biomechanical, neurophysiological, psychological, and other nonspecific mechanisms are stimulated via MT to improve musculoskeletal and respiratory outcomes [11] in patients with NSCNP. Many systematic reviews analyze the effectiveness of MT in musculoskeletal outcomes [10], but there is a deficit in the existing literature regarding the effectiveness of MT in respiratory outcomes. After a systematic search in the scientific databases of PubMed, PEDro, and Scopus, we identified only one previous systematic review that addressed mostly the association between respiratory outcomes and NSCNP [12]. However, no systematic reviews have so far addressed the effectiveness of interventions targeting the respiratory muscles for NSCNP relief.

Based on these findings and the hypotheses underlying the observed respiratory dysfunction in patients with NSCNP, the aim of this systematic review was to address the following questions:

(1) What is the combined or relative effectiveness of MT, according to the definition of IFOMPT, in the form of (a) spinal and diaphragmatic manual therapy, (b) specific neck stabilizing exercises, and (c) breathing re-education exercises of the respiratory muscles on musculoskeletal and respiratory outcomes in patients with NSCNP? 

(2) Is there any additional benefit regarding the inclusion of the assessment of respiratory outcomes in monitoring the progress of patients with NSCNP?

## 2. Materials and Methods

This systematic review was conducted according to the Preferred Reporting Items for Systematic Reviews and Meta-Analyses (PRISMA) 2020 guidelines [13], and the methodological quality assessment of the clinical trials was conducted according to the PEDro scale [14,15]. This review has been registered in the PROSPERO database (registration number: CRD42021240397). 

### 2.1. Inclusion and Exclusion Criteria 

Randomized controlled trials (RCTs) published in English were included in the current systematic review that: (a) were relevant to MT intervention, consisting of mobilization or manipulation techniques of the cervical or thoracic spine and/or diaphragm and/or specific stabilization exercises (SE) targeting the deep neck flexor muscles, with or without parallel interventions or studies that were relevant to a respiratory-related intervention (RRI) targeting the respiratory muscles of participants with NSCNP; (b) included either respiratory outcomes and any associated musculoskeletal outcomes (pain, disability) or, in the case that no respiratory outcomes were present, included an intervention targeting the respiratory system; and (c) included only patients with NSCNP aged 18–65 years. 

The current comprehensive review excluded non-randomized controlled clinical trials, studies without control or other intervention groups, trials relevant to the effectiveness of MT primarily in musculoskeletal outcomes, and trials without a respiratory exercise component. Studies were also disqualified if they had people who had any of the following conditions: neck discomfort lasting fewer than six weeks, spinal column operations, fibromyalgia, systemic diseases, neurological disorders, tumors, cancer, chronic obstructive pulmonary disease (COPD), dyspnea, spinal cord injuries, whiplash injuries.

### 2.2. Search Strategy 

From inception to April 2022, a search of electronic databases including PubMed, PEDro (Physiotherapy Evidence Database), Scopus, and one search engine (Google Scholar) was conducted. By hand, additional searches were made in the included studies’ reference lists. The PICO model for clinical questions and other keyword combinations were employed in the Scopus and PEDro database advanced search (Table 1). The algorithm used in the PubMed database (advanced search) is presented as Supplementary Material (Table S1).

Two reviewers (PIT and GAK) independently screened all titles and/or abstracts and reviewed the studies identified for inclusion. Disagreements were resolved by consensus between the two reviewers or by a third reviewer (EG) when required.

### 2.3. Assessment of Methodological Quality 

Using the PEDro scale, two reviewers (PIT and EP) evaluated the methodological quality [14]. The disagreements and results were reevaluated by a third reviewer (ZD). For evaluating the effectiveness of physical therapy and rehabilitation trials, the PEDro scale is a valid and efficient tool [14,15]. On the PEDro scale, there are two items relevant to rating the suitability of statistical requirements and eight items related to internal validity. One point is contributed to the overall PEDro score for each item that is satisfied (0–10 points). No points, however, are given for ambiguous items. The overall [14] as well as the individual [14,15] scores on the PEDro scale items were presented for all studies in total as well as alongside the effectiveness of studies individually addressing each MT treatment component of this review. According to the protocol of previous studies, studies that scored ≥ 6 on the PEDro were classified as studies with high methodological quality [16].

### 2.4. Data Extraction 

Titles and abstracts of the extracted studies were evaluated by one reviewer (PIT). When the abstract did not contain adequate information, the entire article was examined. Data on the year of publication, inclusion–exclusion criteria for individuals, participant demographics (sample size, study population, sex, and age), type, number of sessions, length of each intervention, outcome follow-up, and results of each included study were extracted by two reviewers (PIT and PK) according to PRISMA (Preferred Reporting Items for Systematic Reviews and Meta-Analyses) [13].

### 2.5. Data Synthesis and Analysis 

The Review Manager software (RevMan 5.4) was used to summarize the effects of MT and RRI on pain, disability, and respiratory outcomes. Subgroup analysis was performed for each outcome if there was clinical heterogeneity in the intervention and comparable details of studies. We did not categorize studies based on the follow-up time points, since nearly all included studies (13 out of 16) did not have any additional follow-up periods apart from those at the end of the intervention period. 

Quantitative synthesis was carried out in accordance with the recommendations in the Cochrane Handbook for Systematic Reviews of Interventions, using the pre-post means and standard deviations from each chosen study for the between-group comparisons, either extracted directly from the articles or calculated where necessary [17]. Since the studies employed the same outcome assessment procedures for the reported comparisons, the mean difference (MD) and 95% confidence intervals (CI) were used. To determine the clinical relevance of the treatment in each subgroup analysis and overall for each outcome, a random-effects inverse variance model was chosen for meta-analysis. To have a common measure of effect size between different outcomes, standardized mean difference (SMD) values were also presented. The effect size was classified according to Cohen’s criteria as small (≤0.20), moderate (0.21–0.79), and large (≥0.80) [18]. The I^2^ statistic was used to measure heterogeneity, and we interpreted I^2^ values greater than 50% to indicate significant heterogeneity.

A subgroup analysis was conducted depending on the types of intervention used in the assigned groups after studies were pooled primarily according to the outcome of interest.

### 2.6. Quality of Evidence 

We used the Grading of Recommendations Assessment, Development, and Evaluation (GRADE) framework in the meta-analysis for each outcome to assess the overall strength of the recommendations and quality of the evidence [19]. For each serious flaw detected in the following main domains (risk of bias, inconsistency, indirectness, imprecision, publication bias), meta-analyses of studies that were initially rated as high-quality evidence were lowered by one level. Specifically, for the risk of bias, we downgraded the quality of the evidence when more than 25% of the studies in each comparison were deemed to be of low quality; for inconsistency, we downgraded the quality of the evidence when significant heterogeneity (I^2^ value > 50%) and/or minimal or no overlap of confidence intervals and wide variance of point estimates across studies were detected; for indirectness, we downgraded the quality of the evidence when participants, interventions, or outcome measures between included studies were significantly different; for imprecision, the quality of evidence was lowered when the pooled sample had 400 participants or fewer and/or wide confidence intervals were apparent, indicating a lack of benefit of the investigated intervention; and for publication bias, we lowered the quality of evidence in meta-analyses that included > 10 studies that presented with asymmetrical funnel plots, sponsored studies, and/or studies where authors declared a conflict of interest. The quality of the evidence was rated as extremely poor, low, moderate, or high based on the aforementioned criteria.

## 3. Results

### 3.1. Identification of Studies

**Figure 1:**
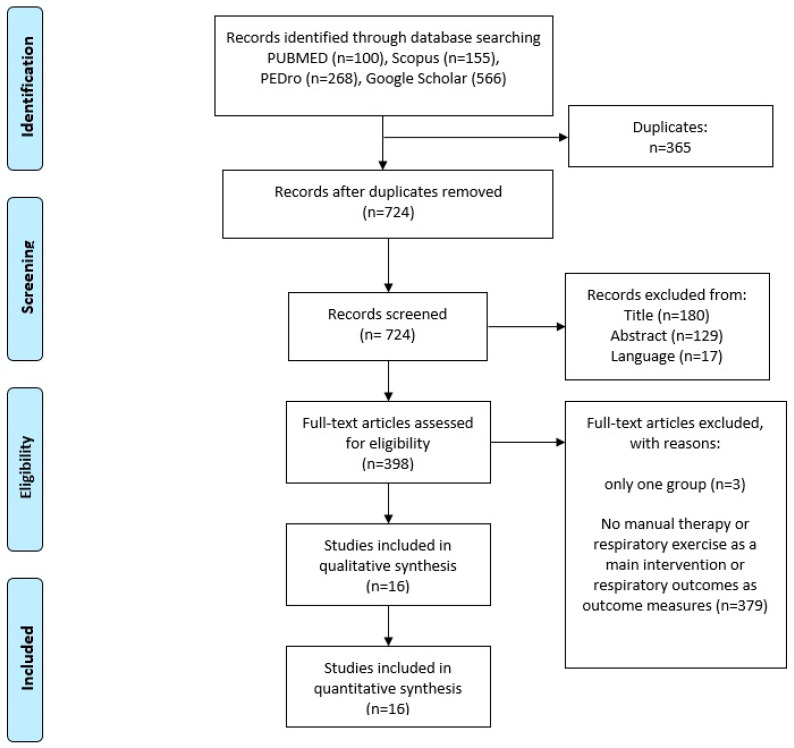
PRISMA flowchart showing the selection procedure for the studies in this systematic review.

The electronic search identified 1089 studies. A total of 100 RCTs were collected from PubMed, 155 from Scopus, 268 from the PEDro database, and 566 from Google Scholar. A number of studies (*n* = 562) were excluded (365 were duplicates, 17 in a language other than English, and 180 from the title). The remaining 527 studies were screened and 129 excluded after studying the abstract. The remaining 398 articles were obtained for inspection in full text, and another 382 articles were excluded based on the inclusion and exclusion criteria of the present systematic review. A total of 16 RCTs were finally included in the present systematic review. A detailed flowchart is provided in Figure 1.

### 3.2. Methodological Quality

On average, the methodological quality score of all included studies rated with the PEDro scale (Table 2) was 5.62/10. Specifically, only one study was rated 9/10 [20], two 8/10 [21,22], five 6/10 [23,24,25,26,27], three 5/10 [28,29,30], and five 4/10 [31,32,33,34,35]. 

**Figure 2:**
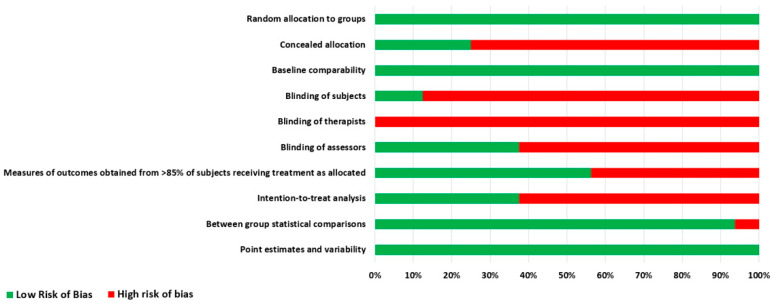
Resulting risk of bias per methodological quality item assessed with the PEDro scale.

When the 10 components of the PEDro scale were examined individually, the following six categories—concealed allocation, subject blinding, therapist and assessor blinding, adequate follow-up, and as-planned/intention-to-treat analysis—presented as significant sources of bias. More than or equal to 50% of publications did not address these sources of bias (Figure 2). 

### 3.3. Participants

Participant details are presented as Supplementary Material (Table S2). All the included studies mentioned the exact number of participants allocated to each group, their age, the sex distribution of participants (except for two studies [22,34]), their BMI (except for one study [23]), and the duration of symptoms. With the exception of two trials, 14 of the 16 included studies conducted an a priori sample size calculation to attain a power level of at least 80% at a significance level of 0.05 for one of their primary outcome measures [29,32].

### 3.4. Interventions and Control Group Content

In all included studies, the experimental group included ΜΤ as an intervention in the form of joint [20,21,23,25,28] and/or soft tissue mobilization/manipulation at the cervical or thoracic spine or diaphragm [20,21,23,28] or in the form of specific exercise of the deep neck flexor muscles [22,23,24,26,30,31,33,34] that contribute to spinal stabilization. Exercises contributing to the re-education of the respiratory muscles [21,22,23,27,32,35] were also considered as experimental group intervention. These interventions were compared with other therapeutic approaches (traditional physiotherapy, exercise, therapeutic patient education) or a true control group (wait and see, sham or no intervention). The duration of interventions ranged from 1 day to 8 weeks. Details of the studies are analytically provided (Table S2).

### 3.5. Intervention Comparability

All of the included studies were randomized, with a control group and an adequate number of individuals. Four studies had a relatively small number of participants per group: 4–10 participants per group [22,31,32,33].

Although significant clinical heterogeneity was noted between the included studies—attributed to: (a) differences in the techniques utilized targeting the spinal joints, trunk, or respiratory muscles; and variability in (b) intervention duration and (c) the outcomes assessed between studies, a quantitative synthesis was also performed where possible. 

### 3.6. Effects of Interventions

#### 3.6.1. Effects of MT on Pain 

The effects of single joint mobilization/manipulation or diaphragm MT on pain were not reported in any of the included studies. 

**Figure 3:**
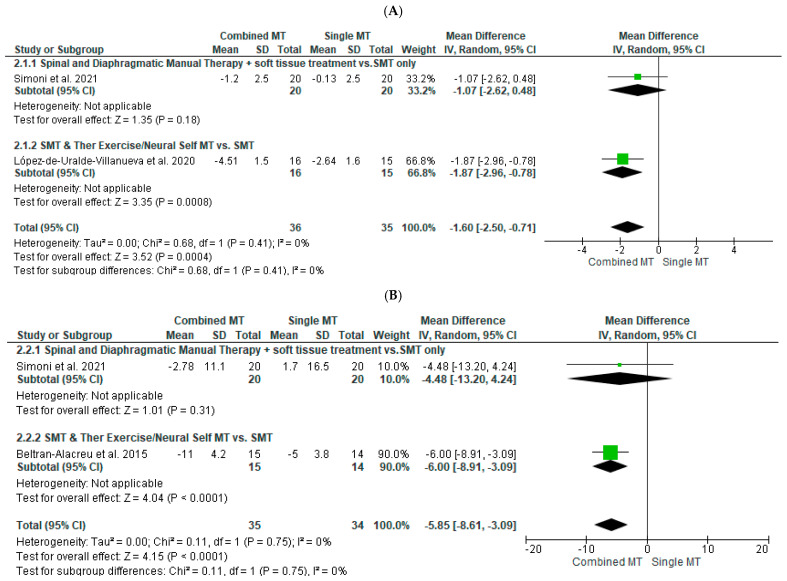
Forest plot showing the effects of combined compared to single manual therapy techniques with or without other parallel interventions on (**A**) pain (references [20,21]) and (**B**) disability (references [20,23]).

The effects of combined—compared to single—MT techniques, with or without other parallel interventions, on pain were evaluated by two studies [20,21] including 71 participants in total (Figure 3A). A moderate effect size [SMD (95% CI) = −0.76 (−1.5 to −0.03), favoring combined MT] with statistical significance (*p* < 0.001) and no statistical heterogeneity was noted, based, however, on low-quality evidence (Table 3A). 

**Figure 4:**
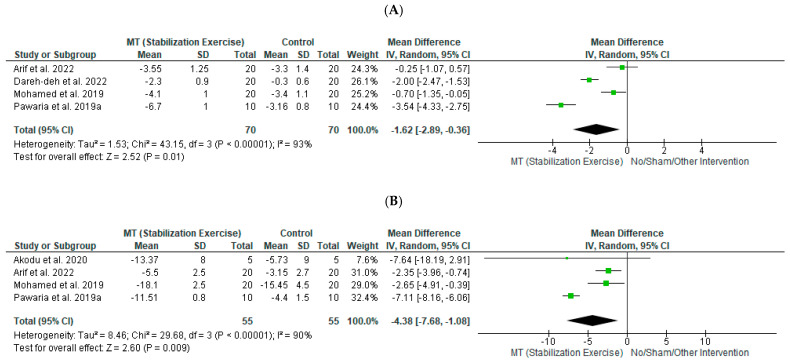
Forest plot showing the effects of stabilization exercise manual therapy with or without other interventions compared to no or sham treatment or other interventions on (**A**) pain (references [22,26,30,33]) and (**B**) disability (references [26,30,31,33]).

The effects of SE with conventional physiotherapy compared to conventional physiotherapy on pain were evaluated by four studies [22,26,30,33] including 140 participants in total (Figure 4A). A large effect size [SMD (95% CI) = −1.65 (−2.99 to −0.31), favoring SE] with statistical significance (*p* = 0.01) was noted but with high statistical heterogeneity (I^2^ = 93%) and based on very low-quality evidence (Table 3C).

#### 3.6.2. Effects of MT on Disability

The effects of single joint mobilization/manipulation or diaphragm MT on disability were not reported in any of the included studies. 

The effects of combined—compared to single—MT techniques, with or without other parallel interventions, on disability were evaluated by two studies [20,23] including 69 participants in total (Figure 3B). A large effect size [SMD (95% CI) = −0.85 (−1.5 to −0.03), favoring combined MT] with statistical significance (*p* < 0.001) and no statistical heterogeneity was noted, based, however, on low-quality evidence (Table 3A). 

The effects of SE with conventional physiotherapy compared to conventional physiotherapy on disability were evaluated by four studies [26,30,31,33] including 110 participants in total (Figure 4B). A large effect size [SMD (95% CI) = −1.6 (−2.83 to −0.37), favoring SE] with statistical significance (*p* = 0.009) was noted but with high statistical heterogeneity (I^2^ = 90%) and based on very low-quality evidence (Table 3C).

#### 3.6.3. Effects of MT on Respiratory Outcomes

The effects of single neck joint mobilization/manipulation or diaphragm MT on respiratory outcomes were not reported in any of the included studies. 

**Figure 5:**
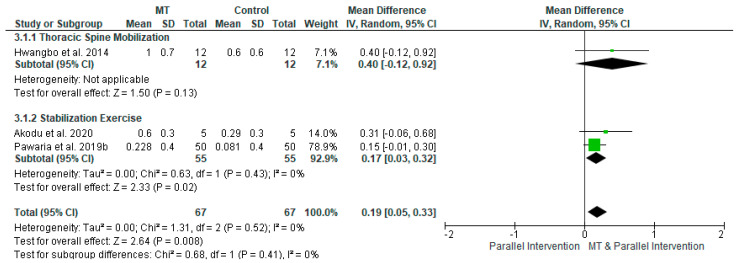
Forest plot showing the effects of manual therapy (thoracic spinal manual therapy or stabilization exercise) with parallel intervention compared to parallel intervention only on FEV1 (references [28,31,34]).

The effects of single MT techniques (thoracic spinal MT or SE) with other parallel interventions compared to parallel interventions only on FEV1 were evaluated by three studies [28,31,34] including 134 participants in total (Figure 5). A moderate effect size [SMD (95% CI) = 0.44 (0.1 to 0.79), favoring MT] with statistical significance (*p* = 0.008) and no statistical heterogeneity was noted, based, however, on very low-quality evidence (Table 3B). 

**Figure 6:**
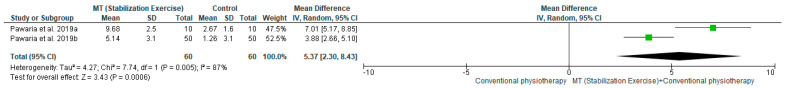
Forest plot showing the effects of stabilization exercise manual therapy with conventional physiotherapy compared to conventional physiotherapy treatment only on maximum inspiratory pressure (references [33,34]).

The effects of SE with conventional physiotherapy compared to conventional physiotherapy on maximum inspiratory pressure (PImax) were evaluated by two studies [33,34] including 120 participants in total (Figure 6). A large effect size [SMD (95% CI) = 2.1 (0.2 to 4.0), favoring SE] with a statistical significance (*p* < 0.001) was noted but with high statistical heterogeneity (I^2^ = 87%) and based on very low-quality evidence (Table 3C).

#### 3.6.4. Effects of RRI on Pain 

**Figure 7:**
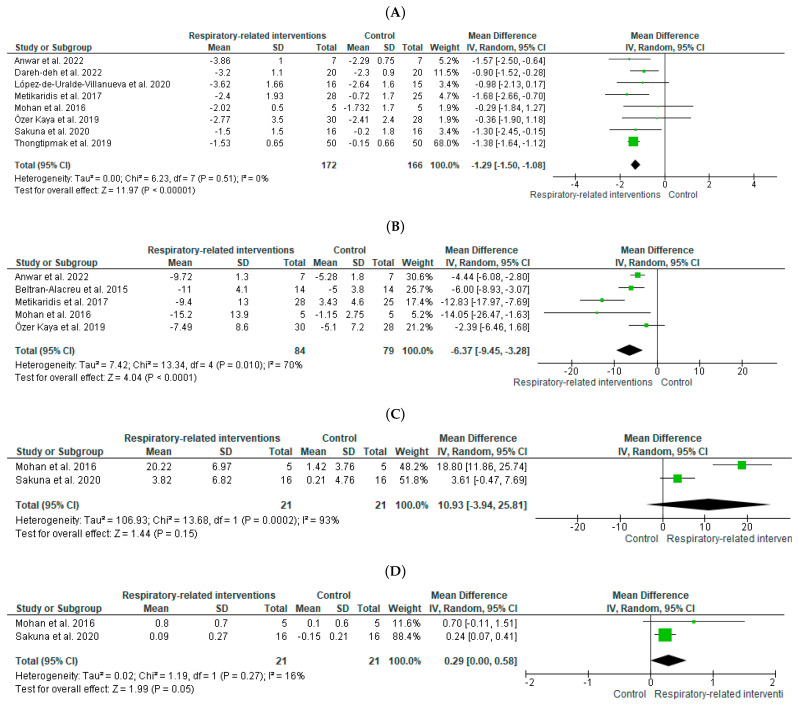
Forest plot showing the effects of respiratory-related interventions with or without other parallel interventions compared to control or other parallel interventions only on (**A**) pain (references [21,22,24,25,27,29,32,35]), (**B**) disability (references [23,24,27,29,32]), (**C**) maximum voluntary ventilation (references [25,32]), and (**D**) chest wall expansion (references [25,32]).

The effects of RRI with or without other parallel interventions—compared to control or other parallel interventions—on pain were evaluated by eight studies [21,22,24,25,27,29,32,35] including 338 participants in total (Figure 7A). A large effect size [SMD (95% CI) = −0.91 (−1.45 to −0.36), favoring RRI] with statistical significance (*p* < 0.001) and no statistical heterogeneity was noted, based, however, on low-quality evidence (Table 3D). 

#### 3.6.5. Effects of RRI on Disability 

The effects of RRI with or without other parallel interventions—compared to control or other parallel interventions—on disability were evaluated by five studies [23,24,27,29,32] including 163 participants in total (Figure 7B). A large effect size [SMD (95% CI) = −1.22 (−1.92 to −0.52), favoring RRI] with statistical significance (*p* = 0.01) was noted but with high statistical heterogeneity (I^2^ = 70%) and based on very low-quality evidence (Table 3D). 

#### 3.6.6. Effects of RRI on Respiratory Outcomes 

The effects of RRI with or without other parallel interventions—compared to control or other parallel interventions—on maximum voluntary ventilation (MVV) and chest wall expansion (CWE) were evaluated by two studies [25,32] including 42 participants in total (Figure 7C,D). A large effect size was noted for MVV [SMD (95% CI) = 1.6 (0.74 to 3.95), favoring RRI] but without statistical significance (*p* = 0.15), with high statistical heterogeneity (I^2^ = 93%), and based on very low-quality evidence (Table 3). Similarly, a large effect size was noted for CWE [SMD (95% CI) = 0.97 (0.32 to 1.62), favoring RRI] but with no statistical significance (*p* = 0.05) and no statistical heterogeneity and based on very low-quality evidence (Table 3D). 

## 4. Discussion

### 4.1. Clinical Considerations

The effectiveness of mobilizing/manipulating the diaphragm, according to Chaitow [36], and the cervical or thoracic spine, according to the Maitland [37] or Kaltenborn concepts of joint manual therapy [38]; core stability exercises (SE); and respiratory-related interventions (RRI) in patients with NSCNP were examined in the current systematic review. Different combinations of the interventions were examined in relation to other interventions or sham techniques. 

Different mobilization/manipulation strategies were applied to the experimental groups, adopting various combinations, methods, and techniques according to the Kaltenborn [28], Maitland [21,23], and osteopathic treatment [20] concepts (and one study [25] did not report the exact technique of manual therapy utilized). These techniques were applied at different vertebral segments: cervical [20,21,23] and/or thoracic [21,23,25,28], and/or the diaphragm [20]. In one trial, mobilization and manipulation techniques served as the sole intervention [28] at the thoracic spine. These techniques were applied in another group in combination with self-stretching exercise (Group C). Mobilization/manipulation techniques of the cervical spine were applied in combination with diaphragm manual therapy in one study and compared with the same cervical spine mobilization/manipulation techniques in combination with sham diaphragm manual therapy as a control group [20]. One study [25] did not mention the exact chest wall mobilization technique methodology used. 

All of the above studies demonstrate the lack of existing clinical trials in examining the effectiveness of diaphragmatic manual techniques, as only one relevant study was identified as having this aim [20]. Additionally, there was a significant clinical heterogeneity between the included studies regarding mobilization/manipulation delivery. Further, there was no clinical trial examining the effect of cervical mobilization techniques as a single intervention on respiratory outcomes in patients with NSCNP.

Importantly, all of the included clinical trials utilized exercise as a part of treatment in either the intervention or the control group. Specifically, diaphragmatic breathing re-education exercise was proposed to confer benefits under mechanisms of psychosomatic relaxation or via lymphatic system activation, homeostasis of the functions of the whole body, and autonomous nervous system recalibration [21,22,23,24,25,27,29,32,35]. Only one clinical trial examined the effect of respiratory exercises on the endurance characteristics of the muscles involved [32]. Furthermore, the experimental groups received different dosages of respiratory exercises in the form of diaphragmatic breathing re-education exercises [21,23,24,25,29,32], pursed lips breathing exercises [32], diaphragm relaxation exercises [21,29], deep slow breathing [35], respiratory exercises with the use of a spirometer [32], respiratory exercises with the use of a balloon [22], and respiratory exercises focusing on proper inhalation, exhalation, and chest expansion [27]. RRIs were delivered in different combinations: in one study, as the only intervention [29] compared with no intervention; in three studies, combined with spinal manual therapy [21,23,25] and compared with spinal manual therapy in combination with therapeutic exercise and neural self-mobilization [21,23] and with no intervention [25]; and in one study, combined with therapeutic exercises [22] and compared with a pamphlet including information on postural corrections and improving general health. Respiratory exercises in two studies were combined with routine physiotherapy [27,32] and compared with routine physiotherapy [27,32]. Additionally, respiratory exercises in three studies were combined with cervical stabilization exercises [22,24,35] and, in two of the studies, compared with cervical stabilization exercises [22,24]; in the other one, they were compared with no intervention [35]. Consequently, respiratory exercises have been prescribed in various combinations and dosages, and, for many clinical trials, these were not delivered as the main intervention.

Conversely, stabilization exercises of the deep cervical flexors were applied in combination with conventional physiotherapy in five studies [26,30,31,33,34], two of them by the same author [33,34], four compared with conventional physiotherapy as a control condition [26,30,33,34], and one compared with neck stabilization exercises and with isometric exercises [31]. Although this type of exercise seems to have a positive effect on respiratory outcomes in patients with NSCNP, possibly via improvements in motor control of the neck muscles, in forward head posture, in the thoracic curve, and in chest expansion, the studies that included stabilization exercise were of poor methodological quality. 

### 4.2. Effectiveness According to Different Therapeutic Approaches

In general, several gaps in the literature presented were identified. The effect of cervical spine mobilization/manipulation MT on a combination of outcomes (pain, disability, and respiratory outcomes) could not be evaluated in unison. Quantitative analyses were presented via clinically meaningful combinations of studies; however, in general, these did not include a large number of studies/participants, presented statistical heterogeneity, and were based on low- to very low-quality evidence. 

The current systematic review is the only one that has been completed that considers MT as defined by IFOMPT. It also specifically focuses on respiratory exercises and diaphragmatic release mobilization techniques as well as the effects of the aforementioned interventions on NSCNP patients’ musculoskeletal and respiratory clinical outcomes. The majority of systematic reviews consider the effects of MT in the form of core stability exercises and/or joint or soft tissue mobilization techniques and their impact on musculoskeletal outcomes rather than also including respiratory outcomes in patients with NSCNP. The systematic review by Kahlaee et al. [12] examined the association between CNP and respiratory dysfunction, whether respiratory and musculoskeletal clinical outcomes were correlated, and the effect of respiratory retraining on pulmonary function and cervical musculoskeletal outcomes in patients with CNP. Two studies [8,39] were included, and both were not analyzed in the present review, as they did not meet the inclusion criteria. Both of them were single-group cohort studies, without a control or another intervention group. 

The tone of the diaphragm and the local stabilizing muscle system of the spine affect the spine’s core stability in general [7]. Numerous studies support the diaphragm’s role in the local core stability muscle group for efficient stabilization of the spine [7]. Because of inadequate co-contraction and coordination of the core muscles, disruption of motor control results in insufficient control of the joints. Pain reduction related to breathing therapy may involve additional central pain control mechanisms. The vagus nerve, which plays a key role in the transmission and mediation of sensory information between the brain and the peripheral tissues, is stimulated by slow, calm diaphragmatic breathing, according to earlier research. It has been demonstrated that vagus nerve stimulation significantly lowers peripheral inflammatory cytokines; reduces the sympathetic tone; decreases malondialdehyde, which is related to oxidative stress; reduces brain activation patterns related to pain (decreases hippocampus and amygdala activity and increases insular cortical and left prefrontal cortex activity); and controls the effects of opioids [40]. 

This systematic review sought to identify the complex manual therapy procedures administered to NSCNP patients in studies which also addressed the respiratory component, either as a therapeutic intervention or as an outcome, in combination with the musculoskeletal component. Language restrictions (only English-language studies were included), the variety of therapeutic approaches and outcomes, and the inclusion of trials with a wide range of methodological quality and sample size were among this review’s limitations.

## 5. Conclusions

Upon analyzing the results of the current clinical trials, we came to the conclusion that there is currently some emerging evidence that MT (specifically, joint, soft tissue, and diaphragm mobilization/manipulation techniques), core stability exercises supplemented with respiratory exercises, and re-education of diaphragmatic breathing improve musculoskeletal and respiratory outcomes and respiratory function in patients with NSCNP. The results of the current review, however, are subject to the risk of bias, since a number of methodological quality items were not met in many of the included studies. Additionally, there was a great deal of variation in the interventions examined and the outcomes examined; therefore, an initial categorization of these studies was attempted.

It appears that patients with NSCNP would benefit significantly from existing MT programs supplemented with appropriate diaphragm and respiratory exercises. This may be because breathing disorders frequently coexist with or directly impair motor control of all muscles surrounding the spine, and so they should be considered when prescribing exercises for this patient population. The effectiveness of diaphragmatic mobilization/manipulation techniques and respiratory exercises in musculoskeletal and respiratory outcomes calls for additional research investigations of high methodological quality.

## Figures and Tables

**Table 1:** The PICO model applied to this systematic review.

**Patient/Client** **G** **roup:**	“neck pain” OR “chronic neck pain” OR “mechanical neck pain”
**Intervention:**	“manual therapy” OR “respiratory exercises” OR “breathing retraining” OR “diaphragm manual therapy” OR “relaxation” OR “diaphragm exercises” AND
**Comparison:**	“control group” OR “traditional physiotherapy” OR “sham” AND
**Outcomes:**	“pain” OR “function” OR “respiratory outcomes” OR “breathing dysfunction” OR “respiratory function” AND
**Study:**	“randomized clinical trial” OR “systematic review”

**Table 2:** Methodological quality of the included studies assessed with the PEDro scale.

Studies	Items										
	1	2	3	4	5	6	7	8	9	10	Total Score
Hwangbo et al., 2014 [28]	+		+				+		+	+	**5/10**
Beltran-Alacreu et al., 2015 [23]	+		+				+	+	+	+	**6/10**
Mohan et al., 2016 [32]	+		+			+				+	**4/10**
Metikaridis et al., 2017 [29]	+		+					+	+	+	**5/10**
Pawaria et al., 2019a [33]	+		+						+	+	**4/10**
Pawaria et al., 2019b [34]	+		+						+	+	**4/10**
Özer Kaya et al., 2019 [24]	+	+	+			+			+	+	**6/10**
Thongtipmak et al., 2019 [35]	+		+						+	+	**4/10**
Mohamed et al., 2019 [30]	+		+				+		+	+	**5/10**
López-de-Uralde-Villanueva et al., 2020 [21]	+	+	+			+	+	+	+	+	**8/10**
Akodu et al., 2020 [31]	+		+						+	+	**4/10**
Sakuna et al., 2020 [25]	+		+			+	+		+	+	**6/10**
Simoni et al., 2021 [20]	+	+	+	+		+	+	+	+	+	**9/10**
Arif et al., 2022 [26]	+		+	+			+		+	+	**6/10**
Anwar et al., 2022 [27]	+		+				+	+	+	+	**6/10**
Dareh-deh et al., 2022 [22]	+	+	+			+	+	+	+	+	**8/10**

**Table 3:** Quality of the evidence in the Grading of Recommendations Assessment, Development, and Evaluation (GRADE) system for outcomes assessed in the meta-analysis. (**A**) Studies examining the effects of combined MT vs. single MT techniques. (**B**) Studies examining the effects of MT (thoracic spinal MT or SEMT) with parallel intervention vs. parallel intervention. (**C**) Studies examining the effects of SEMT with or without other interventions compared to control or other intervention. (**D**) Studies examining the effects of RRI with or without other parallel interventions vs. control or other parallel interventions only.

(**A**)										
**Certainty Assessment**							**No. of Patients**		**Effect**	**Certainty**
**No. of Studies per Outcome**	**Study Design**	**Risk of Bias**	**Inconsistency**	**Indirectness**	**Imprecision**	**Other Considerations**	**Combined MT**	**Single MT**	**Absolute (95% CI)**	
Pain										
2	randomized trials	not serious	not serious	not serious	very serious ^a^	none	36	35	SMD −0.76 (−1.5 to −0.03)	⊕⊕◯◯ Low
Disability										
2	randomized trials	not serious	not serious	not serious	very serious ^a^	none	35	34	SMD −0.85 (−1.96 to −0.27)	⊕⊕◯◯ Low
(**B**)										
**Certainty Assessment**							**No. of Patients**		**Effect**	**Certainty**
**No. of Studies per Outcome**	**Study Design**	**Risk of Bias**	**Inconsistency**	**Indirectness**	**Imprecision**	**Other Considerations**	**MT**	**Parallel Intervention**	**Absolute (95% CI)**	
FEV1										
3	randomized trials	very serious ^a^	not serious	not serious	very serious ^b^	none	67	67	SMD 0.44 (0.1 to 0.79)	⊕◯◯◯ Very low
(**C**)										
**Certainty Assessment**							**No. of Patients**		**Effect**	**Certainty**
**No. of Studies per Outcome**	**Study Design**	**Risk of Bias**	**Inconsistency**	**Indirectness**	**Imprecision**	**Other Considerations**	**SEMT**	**Control/Other Intervention**	**Absolute (95% CI)**	
Pain										
4	randomized trials	serious ^a^	very serious ^b^	not serious	very serious ^c^^,^^d^	none	70	70	SMD −1.65 (−2.99 to −0.31)	⊕◯◯◯ Very low
Disability										
4	randomized trials	very serious ^a^	very serious ^b^	not serious	very serious ^c^^,^^d^	none	55	55	SMD −1.6 (−2.83 to −0.37)	⊕◯◯◯ Very low
PImax										
2	randomized trials	very serious ^a^	very serious ^b^	not serious	very serious ^c^^,^^d^	none	60	60	SMD 2.1 (0.2 to 4)	⊕◯◯◯ Very low
(**D**)										
**Certainty Assessment**							**No. of Patients**		**Effect**	**Certainty**
**No. of Studies per Outcome**	**Study Design**	**Risk of Bias**	**Inconsistency**	**Indirectness**	**Imprecision**	**Other Considerations**	**RRI**	**Control/Other Intervention**	**Absolute (95% CI)**	
Pain										
8	randomized trials	serious ^a^	not serious	not serious	serious ^b^	none	172	166	SMD −0.91 (−1.45 to −0.36)	⊕⊕◯◯ Low
Disability										
5	randomized trials	serious ^a^	serious ^c^	not serious	serious ^b^	none	84	79	SMD −1.22 (−1.92 to −0.52)	⊕◯◯◯ Very low
MVV										
2	randomized trials	serious ^a^	very serious ^c^	not serious	very serious ^b^	none	21	21	SMD 1.6 (0.74 l to 3.95)	⊕◯◯◯ Very low
CWE										
2	randomized trials	serious ^a^	not serious	not serious	very serious ^b^	none	21	21	SMD 0.97 (0.32 to 1.62)	⊕◯◯◯ Very low

(**A**) **CI:** confidence interval; **SMD:** standardized mean difference; **MT:** manual therapy; ^a^: sample < 400. (**B**) **CI:** confidence interval; **SMD:** standardized mean difference, **MT:** manual therapy; **SEMT:** stabilization exercise manual therapy; ^a^: more than 25% of the studies included are classified as having high risk of bias (<6/10 on PEDro scale total score); ^b^: sample < 400. (**C**) **CI:** confidence interval; **SMD:** standardized mean difference; **SEMT:** stabilization exercise manual therapy; **PImax:** maximum inspiratory pressure; ^a^: more than 25% of the studies included are classified as having high risk of bias (<6 PEDro scale total score); ^b^: minimal or no overlap of CI; heterogeneity (*p* < 0.05); I^2^ > 50%; ^c^: sample < 400; ^d^: wide confidence interval. (**D**) **CI:** confidence interval; **SMD:** standardized mean difference; **RRI:** respiratory-related interventions; ^a^: more than 25% of the studies included are classified as having high risk of bias (<6 on PEDro scale total score); ^b^: sample < 400; ^c^: minimal or no overlap of CI; heterogeneity (*p* < 0.05); I^2^ > 50%. Certainty of the evidence rating: ⊕◯◯◯ Very low; ⊕⊕◯◯ Low.

## Data Availability

Not applicable.

## Supplementary Materials

The following supporting information can be downloaded at: https://www.mdpi.com/article/10.3390/diagnostics12071598/s1, Table S1: PubMed algorithm used in the current study. Table S2: Characteristics of randomized controlled trials in the systematic review.
