# Supplementary material for: The Effectiveness of Spinal, Diaphragmatic, and Specific Stabilization Exercise Manual Therapy and Respiratory-Related Interventions in Patients with Chronic Nonspecific Neck Pain: Systematic Review and Meta-Analysis
Source: Diagnostics (Basel). 2022 Jun 30;12(7):1598. doi: 10.3390/diagnostics12071598 (PMC9316964; doi:10.3390/diagnostics12071598)
Supplement: Supplementary file 1 [file diagnostics-12-01598-s001.zip › Table S1.pdf]

Table S1. PubMed algorithm used in the current study (search performed on 30/04/2022)

Search number	Search details	Results	Time
22	#3 AND #10 AND #14 AND #21,,,"(""neck pain""[Title/Abstract] OR ""chronic neck pain""[Title/Abstract]) AND (((""diaphragm""[MeSH Terms] OR ""diaphragm""[All Fields] OR ""contraceptive devices, female""[MeSH Terms] OR (""contraceptive""[All Fields] AND ""devices""[All Fields] AND ""female""[All Fields]) OR ""female contraceptive devices""[All Fields] OR ""diaphragms""[All Fields] OR ""diaphragm s""[All Fields] OR ""diaphragmal""[All Fields]) AND ""manual""[Title/Abstract]) OR ""respiratory exercises""[Title/Abstract] OR ""breathing retraining""[Title/Abstract] OR ""manual therapy""[Title/Abstract] OR ((""diaphragm""[MeSH Terms] OR ""diaphragm""[All Fields] OR ""contraceptive devices, female""[MeSH Terms] OR (""contraceptive""[All Fields] AND ""devices""[All Fields] AND ""female""[All Fields]) OR ""female contraceptive devices""[All Fields] OR ""diaphragms""[All Fields] OR ""diaphragm s""[All Fields] OR ""diaphragmal""[All Fields]) AND ""mobilization""[Title/Abstract]) OR ""relaxation""[Title/Abstract]) AND (""control group""[Title/Abstract] OR ""sham""[Title/Abstract] OR ""physiotherapy""[Title/Abstract]) AND (""respiratory outcomes""[Title/Abstract] OR ""respiratory function""[Title/Abstract] OR ""chest wall""[Title/Abstract] OR ""breathing dysfunction""[Title/Abstract] OR ""pain""[Title/Abstract] OR ""NDI""[Title/Abstract])"	100	07:18:54
21	#15 OR #16 OR #17 OR #18 OR #19 OR #20,,,"""respiratory outcomes""[Title/Abstract] OR ""respiratory function""[Title/Abstract] OR ""chest wall""[Title/Abstract] OR ""breathing dysfunction""[Title/Abstract] OR ""pain""[Title/Abstract] OR ""NDI""[Title/Abstract]"	724,598	07:03:47
20	NDI[Title/Abstract],,,"""NDI""[Title/Abstract]"	3,684	07:03:13
19	pain[Title/Abstract],,,"""pain""[Title/Abstract]"	689,497	07:03:04
18	breathing dysfunction[Title/Abstract],,,"""breathing dysfunction""[Title/Abstract]"	47	07:02:55
17	chest wall[Title/Abstract],,,"""chest wall""[Title/Abstract]"	18,410	07:01:37
16	respiratory function[Title/Abstract],,,"""respiratory function""[Title/Abstract]"	14,714	07:01:24
15	respiratory outcomes[Title/Abstract],,,"""respiratory outcomes""[Title/Abstract]"	2,517	07:01:05

14	#11 OR #12 OR #13,,,"""control group""[Title/Abstract] OR ""sham""[Title/Abstract] OR ""physiotherapy""[Title/Abstract]"	553,427	06:56:23
13	physiotherapy[Title/Abstract],,,"""physiotherapy""[Title/Abstract]"	22,283	06:56:01
12	sham[Title/Abstract],,,"""sham""[Title/Abstract]"	92,567	06:55:55
11	control group[Title/Abstract],,,"""control group""[Title/Abstract]"	449,688	06:55:49
10	#4 OR #5 OR #6 OR #7 OR #8 OR #9,,,"((""diaphragm""[MeSH Terms] OR ""diaphragm""[All Fields] OR ""contraceptive devices, female""[MeSH Terms] OR (""contraceptive""[All Fields] AND ""devices""[All Fields] AND ""female""[All Fields]) OR ""female contraceptive devices""[All Fields] OR ""diaphragms""[All Fields] OR ""diaphragm s""[All Fields] OR ""diaphragmal""[All Fields]) AND ""manual""[Title/Abstract]) OR ""respiratory exercises""[Title/Abstract] OR ""breathing retraining""[Title/Abstract] OR ""manual therapy""[Title/Abstract] OR ((""diaphragm""[MeSH Terms] OR ""diaphragm""[All Fields] OR ""contraceptive devices, female""[MeSH Terms] OR (""contraceptive""[All Fields] AND ""devices""[All Fields] AND ""female""[All Fields]) OR ""female contraceptive devices""[All Fields] OR ""diaphragms""[All Fields] OR ""diaphragm s""[All Fields] OR ""diaphragmal""[All Fields]) AND ""mobilization""[Title/Abstract]) OR ""relaxation""[Title/Abstract]"	129,049	06:55:21
9	relaxation[Title/Abstract],,,"""relaxation""[Title/Abstract]"	125,791	06:54:54
8	Diaphragm mobilization[Title/Abstract],,,"(""diaphragm""[MeSH Terms] OR ""diaphragm""[All Fields] OR ""contraceptive devices, female""[MeSH Terms] OR (""contraceptive""[All Fields] AND ""devices""[All Fields] AND ""female""[All Fields]) OR ""female contraceptive devices""[All Fields] OR ""diaphragms""[All Fields] OR ""diaphragm s""[All Fields] OR ""diaphragmal""[All Fields]) AND ""mobilization""[Title/Abstract]"	199	06:54:38
7	manual therapy[Title/Abstract],,,"""manual therapy""[Title/Abstract]"	2,705	06:54:24
6	breathing retraining[Title/Abstract],,,"""breathing retraining""[Title/Abstract]"	161	06:54:06
5	respiratory exercises[Title/Abstract],,,"""respiratory exercises""[Title/Abstract]"	146	06:54:02

4	diaphragm manual[Title/Abstract],,,"(""diaphragm""[MeSH Terms] OR ""diaphragm""[All Fields] OR ""contraceptive devices, female""[MeSH Terms] OR (""contraceptive""[All Fields] AND ""devices""[All Fields] AND ""female""[All Fields]) OR ""female contraceptive devices""[All Fields] OR ""diaphragms""[All Fields] OR ""diaphragm s""[All Fields] OR ""diaphragmal""[All Fields]) AND ""manual""[Title/Abstract]"	170	06:53:52
3	#1 OR #2,,,"""neck pain""[Title/Abstract] OR ""chronic neck pain""[Title/Abstract]"	10,957	06:48:26
2	chronic neck pain[Title/Abstract],,,"""chronic neck pain""[Title/Abstract]"	1,200	06:48:14
1	neck pain[Title/Abstract],,,"""neck pain""[Title/Abstract]"	10,957	06:48:09
