# Supplementary material for: The Effectiveness of Spinal, Diaphragmatic, and Specific Stabilization Exercise Manual Therapy and Respiratory-Related Interventions in Patients with Chronic Nonspecific Neck Pain: Systematic Review and Meta-Analysis
Source: Diagnostics (Basel). 2022 Jun 30;12(7):1598. doi: 10.3390/diagnostics12071598 (PMC9316964; doi:10.3390/diagnostics12071598)
Supplement: Supplementary file 1 [file diagnostics-12-01598-s001.zip › Table S2.pdf]

Table S2. Characteristics of randomized controlled trials in the systematic review.

STUDY	METHODOLOGY	CRITERIA	INTERVENTIONS	OUTCOME MEASURES	MAIN RESULTS
**Hwangbo et al. 2014 [28]**<br><br>**Aim:** To determine the effect of thoracic joint mobilization and self-stretching exercise on pulmonary function of patients with chronic neck pain.	Randomized clinical trial,<br><br>Participants: 34 (Female:17, Male:17), Age: A)55.6±1.1years, B) 53.0±0.8 years, and C) 54.4±0.6 years, Height: A) 164.5±2.6 cm, B) 166.8±2.8 cm, C) 167.3±2.3 cm, Weight: A) 63.6±3.5 kg B) 66.8±2.8 kg C) 65.0±3.5 kg<br><br>Duration: 6 weeks (40ˈ/day, 3 days/week)	**Inclusion criteria:**<br>i) nonspecific and mechanical CLBP,<br>ii) patients with thoracic breathing method<br><br>**Exclusion criteria:**<br>i) previous orthopedic and neurosurgical surgery,<br>ii) cardiovascular disease and high risk of falls,<br>iii) other chronic pain,<br>iv) participation in other exercise programs (abdominal muscle training within 1 year),<br>v) pregnancy within 2 years prior,<br>vi) malignant tumor, and<br>vii) radiating pain at two sites	**Intervention group A (n=11):** Thoracic joint mobilization group (TJMG) according to Kaltenborn 15ˈ/session<br><br>**Intervention group B (n=12)**: self-streching exercise group (SSEG) 15ˈ/session<br><br>**Intervention group C (n=12)**:<br>TJM plus SSE (TJMSSEG) 30ˈ/session	**Pulmonary functions**: forced vital capacity (FVC), forced expiratory volume at one second (FEV1), and peak expiratory flow (PEF) were measured using Cardio Touch equipment.<br><br>All outcomes evaluated at baseline, at 4 and 6 weeks of treatment<br><br>*A priori sample size calculation performed for MIP (primary outcome) to detect a between-group significant difference (a=0.05) and power of 80%.*	Comparisons within each of the groups showed that FVC, FEV1, and PEF increased significantly ($p < 0.05$).<br>Among the study groups, FVC was significantly higher in TJMSSEG than in TJMG after six weeks; FEV1 was significantly higher in TJMSSEG than in TJMG and SSEG after four and six weeks ($p < 0.05$); and PEF was significantly higher in TJMSSEG than in TJMG and SSEG after six weeks ($p <0.05$).
**Beltran-Alacreu et al. 2015 [23]**<br><br>**Aim:** To determine the effectiveness of a multimodal treatment in the short and medium term for disability in nonspecific chronic neck pain.	Randomized control trial,<br><br>Participants: 45, (Female:37, Male:9), Age: 18-65years, BMI-,<br><br>Duration: 4 weeks (2 days/week)	**Inclusion criteria:**<br>i) neck pain for at least 12 weeks<br>ii) they understood, wrote, and spoke Spanish fluently<br>iii) pain localized in the neck region<br><br>**Exclusion criteria:**<br>i) neck pain associated with whiplash injuries,	**Control group (n=15):** manual therapy and high-velocity technique in the thoracic dorsal region 25ˈ/session<br><br>**Intervention group A (n=15):** the same treatment as the control group plus therapeutic patient education (including	**Function:** NDI,<br>**Psychology status:** TSK,<br>**Patient beliefs:** FABQ,<br>**Muscle Endurance and sensation of fatigue:** NFME and VAFS<br><br>All outcomes evaluated at baseline, at 4 weeks of treatment, and at 8 and 16 weeks	NDI showed statistically significant differences between baseline outcomes and all follow-up periods ($P< 0.01$).<br>Differences were found for the VAFS and the NFME in the follow-ups at 8 and 16 weeks ($P<0.05$).

		ii) medical red flag history (i.e., tumor, fracture, metabolic diseases, rheumatoid arthritis, and osteoporosis),<br>iii) neck pain with cervical radiculopathy,<br>iv) neck pain associated with externalized cervical disc herniation,<br>v) fibromyalgia syndrome,<br>vi) previous neck surgery, or neck pain accompanied by vertigo caused by vertebrobasilar insufficiency or accompanied by non-cervicogenic headaches	diaphragmatic breathing) in two sessions<br>25ˈ+20ˈ/session<br><br>**Intervention group B (n=15):**<br>The same treatment as control plus intervention A groups plus a progressive therapeutic exercise protocol with stabilization exercises of the cervical region and neural self-mobilization.<br>Patients were asked to perform the exercises at least once per day at home for the next 8 wks.	*A priori sample size calculation performed for NDI (primary outcome) to detect a between-group moderate effect size (d=0.25), significance level (a=0.05) and power of 90%.*	Analysis of variance for group x time interaction showed statistically significant changes (TSK), F = 3.613, P = 0.005; FABQ, F = 2.803, P =0.022).<br>Minimal detectable changes were obtained in both experimental groups for the TSK but not in the control group.
**Mohan et al. 2016 [32]**<br><br>**Aim:** To determine the effects of respiratory exercises on respiratory muscle endurance, cervical range of motion and chest expansion respiratory exercises in CNP.	Randomized clinical trial, pilot<br><br>Participants: 10, (Female:8, Male:2), Age: 22-79 years, BMI: 25.94 ±3.18 kg/m2<br>Height:157.80±9.43cm<br><br>Duration: 8 weeks (40ˈ/session, 2 days/week)	**Inclusion criteria:**<br>i) history of CNP with or without headache for more than three months and<br>ii) free from respiratory and cardiac illness<br><br>**Exclusion criteria:**<br>i) subjects who had a past history of surgery to the cervical spine and<br>ii) those who participated in physiotherapy exercise program	**Intervention group (n=5):**<br>Respiratory exercises plus routine physiotherapy (Diaphragmatic Breathing exercises, VODIS and pursed lip breathing exercises)<br><br>**Control group (n=5):**<br>Routine physiotherapy exercises	**Function:** NDI<br>**Pain:** CN-VAS<br>**Cervical range of motion:** universal goniometer<br>**Chest wall expansion**: cloth tape at axilla, fourth intercostal space and xiphoid level height.,<br>**Respiratory outcomes**: hand held spirometry, MVV<br><br>All outcomes evaluated at baseline and at 8 weeks of treatment	Highly significant increase in MVV from before (mean 34.88, SD 21.81) to after treatment (mean 55.10, SD 16.76 and t-value 6.48) with P = 0.003 in the intervention group vs control group from before (mean 38.32, SD 19.50) to after treatment (mean 39.74, SD 17.56 and t-value 0.845) with P = 0.446. Scores in the active flexion and for the VAS also showed

					significant difference in the intervention group P< 0.05.
**Metikaridis et al 2017 [29]**<br>**Aim**: To examine the effect of a simple, zero cost stress management program on patients with CNP.	Randomized clinical trial,<br><br>Participants: 53, (Female:50, Male:3), Age: 18-75 years, BMI: 25.94 ±3.18 kg/m2 Height:157.80±9.43cm<br><br>Duration: 8 weeks (40'/session, 2 days/week)	**Inclusion criteria:**<br>i) ages of 18–75, of either gender, ii) have NP in line to Goode et al. definition of Chronic NP ("Prevalence, Practice Patterns and Evidence for Chronic Neck Pain"), iii) have at least twenty- four episodes of NP during the last year (irrespective of duration) and<br>iv) be residents of Athens<br><br>**Exclusion criteria**:<br>i) serious underlying pathology (tumor, infection,<br>ii) serious injury or other non-mechanical cause of NP,<br>iii) clinically significant disc herniation,<br>iv) recent neck-shoulder surgery,<br>v) inflammatory rheumatic disease,<br>vi) severe psychiatric illness,<br>vii) pregnancy,<br>viii) practice of other relaxation techniques and<br>ix) inability to write or read Greek.	**Intervention group (n=28):**<br>program of stress management including: diaphragmatic breathing plus progressive muscle relaxation<br>Total duration of 25'minutes (10' minutes DB and 15'<br>minutes PMR) via an audio CD<br><br>**Control group A (n=25):**<br>no intervention	**Disability:** Neck Pain Disability Index Greek Version (NDI)<br>**Pain Intensity**: VAS<br>**Lifestyle-sociodemographic data**: Everyday Life-Lifestyle-Health Questionnaire (ELHQ)<br>**Stress measurement:** Perceived Stress Scale (PSS)<br>**Anxiety-Depression:** Depression, Anxiety, Stress Scale 21 (DASS21)<br>**Health locus of control**: Health Locus of Control Scale (HLC)<br>**Salivary cortisol measurement:** special devices Salivettes	At the end of the monitoring period, the intervention group showed a statistically significant reduction of stress and anxiety ($p = 0.03$, $p = 0.01$), report of stress related symptoms ($p = 0.003$), percentage of disability due to NP ($p = 0.000$) and NP intensity ($p = 0.002$).<br>At the same time, daily routine satisfaction levels were elevated ($p = 0.019$).<br>No statistically significant<br>difference was observed in cortisol measurements.
**Pawaria et al. 2019a [33]**<br><br>**Aim:** To examine the efficacy of Cervical	Randomized controlled trial,	**Inclusion criteria:**<br>i) with mild neck disability (NDI score 5-15) and<br>ii) who had poor performance (unable to achieve 24 mmHg of	**Intervention group (n=10):**<br>Cervical Stabilization Exercise with the	**Pain**: NRPS,<br>**Function:** NDI,<br>**Forward head posture:** CVA<br>**Respiratory muscle strengths:** MIP and MEP	Significant increase in Craniovertebral angle (from mean value of 37.90 to 59.21) and respiratory strength

Stabilization Exercises on Neck pain, Neck disability, Craniovertebral angle and respiratory muscles strength.	Participants: 20 (Female/Male: not reported<br>Age: 40-49 years,<br>BMI: 22,1 ± 1.5 kg/m²<br><br>Duration: 6 weeks (30ˈ/session,<br>No mention of days/week)	pressure) on Craniocervical flexion test<br><br>**Exclusion criteria:**<br>i) neck pain secondary to trauma,<br>ii) abnormalities/deformities of thoracic region or vertebral column,<br>iii) previous history of any thoracic or vertebral column surgery,<br>iv) BMI >30 and<br>v) history of smoking	conventional Physiotherapy<br><br>**Control group (n=10):**<br>Conventional Physiotherapy	All outcomes evaluated at baseline and at 6 weeks of treatment<br><br>*A priori sample size calculation performed to detect a between-group significant difference (a=0.05), power of 80% and large effect size (d=0.8).*	(PImax from mean value of 65.33 to 75.01, PEmax from mean value of 77.78 to 87.89) with decrease in NPRS (from mean value of 7.6 to 0.90) and NDI (from mean value of 12.11 to 0.60) scores were found in the group that received cervical stabilization exercises as compared to control group ($p<0.05$).
**Pawaria et al. 2019b [34]**<br>Aim: to evaluate the effectiveness of exercises meant for enhancing the stability of the neck with feedback on neck stabilization exercises with feedback in improving the respiratory status.	Randomized clinical trial,<br><br>Participants: 100 (Female: 46/ Male:54),<br>Age: 20-40years,<br>BMI: <30<br><br>Duration: 6 weeks (no mention of days/week)	**Inclusion criteria:**<br>i) chronic neck pain in the Age Group 20-40 years<br>II) with mild neck disability (NDI score 5-15)<br>iii) who had less than optimal performance (unable to attain 24 mm of Hg on Sphygmomanometer) on Craniocervical flexion test<br><br>**Exclusion criteria:**<br>i) reported of neck pain secondary to trauma,<br>ii) abnormalities/ deformities of a thoracic region or vertebral column, iii) previous history of any thoracic or vertebral column surgery,	**Intervention group A (n=50):**<br>Cervical Stabilization Exercise with feedback in addition to routine Physiotherapy treatment.<br><br>**Control group (n=50)**:<br>Regular Physiotherapy treatment<br>isometric exercises, TENS for 30 min (intensity of 10-30 mA, frequency of 80 Hz) and hot packs.<br>All subjects performed Cervical isometric exercises in sitting position by applying resistance at the forehead (cervical flexion,	**FHP:** Craniovertebral Angle (CVA)<br>**Pulmonary function:** Spirometry assessed (FEV1) and Micro RPM assessed inspiratory muscle strength (PImax).<br><br>All outcomes evaluated at baseline and on 3rd and 6th week of treatment<br><br>*A priori sample size calculation performed to detect a between-group significant difference (a=0.05), power of 95% and large effect size (d=0.8).*	Significant reduction in forward head posture measured by improvement in CVA, improvement in Inspiratory muscle strength (PImax) and pulmonary functions (FEV1) were found in the group that received cervical stabilization exercises with feedback along with the conventional physiotherapy ($p< 0.05$).

iv) subjects in the category of overweight as measured by BMI > 30,<br>v) history of smoking	extension, rotation, and side bending) maintained for 10 sec having 15-sec breaks between holds with 10-15 repetitions increasingly

**Özer Kaya et al. 2019 [24]**<br><br>**Aim:** to investigate and compare the effects of a 4-week neck stabilization exercise program plus progressive muscle relaxation training (PMRT) to stabilization exercise program alone in patients with chronic neck pain (CNP)	Randomized clinical trial,<br><br>Participants: 58 (Female/Male: 47/11),<br>Age: 18-65 years,<br><br>Duration: 4 weeks (3 days/week)	**Inclusion criteria:**<br>i) having neck pain for at least 3 to 6 months,<br>ii) being 18 to 65 years of age, and<br>iii) being a volunteer to participate in the study.<br><br>**Exclusion criteria:**<br>i) having disc herniation,<br>ii) spinal stenosis,<br>iii) cervical surgery history,<br>iv) cancer,<br>v) inflammatory rheumatologic diseases,<br>vi) severe psychological disorders,<br>vii) being pregnant,<br>viii) having intervention including exercise program or physiotherapy in the last six months	**Intervention group A (n=28):**<br>Stabilization Exercise<br><br>**Control group (n=30):**<br>Stabilization Exercise plus PMRT according to Jacobson<br>A session lasted about 20min	**Pain intensity:** VAS,<br>**Pressure Pain Threshold (PPT):** Digital Algometer<br>**Range of Motion (ROM):** goniometer,<br>**Disability:** NDI,<br>**Kinesiophobia:** TSK, and<br>**Quality of life:** Short Form-36<br><br>All outcomes evaluated at baseline and on 4th week of treatment<br><br>*A priori sample size calculation performed to detect a between-group significant difference (a=0.05), power of 95% and large effect size (d=0.8).*	Pain intensity, and disability decreased, cervical flexion, extension, right lateral flexion, and rotation movements increased in the Intervention group ($p<0.05$).<br>In the Control group, pain intensity, disability, and kinesiophobia reduced, and pain pressure threshold, all cervical range of movements, and quality of life scores improved ($p<0.05$).<br>Intergroup comparisons showed that the pain pressure threshold, cervical flexion, right lateral flexion, the right and left rotation range of movements, and kinesiophobia improvements were better in the Relaxation Group ($p<0.05$).
**Mohamed et al. 2019 [30]**<br><br>**Aim:** to investigate the effect of deep	Randomized clinical trial,<br><br>Participants: 40 (Female:26 Male:14),	**Inclusion criteria:**<br>i) aged between 20-30 years<br>ii) mechanical neck pain duration from six to<br>twelve months and	**Intervention group (n=20):** Deep cervical flexor muscle training plus the traditional physical therapy program,	**Pain:** VAS<br>**Neck functional disability:** NDI	There was significant improvement in craniocervical flexion test, MVV and PEF

cervical muscle training using pressure biofeedback device on ventilatory functions in patients with chronic mechanical neck pain.	Age: 20-30years,<br>BMI: <30 kg/m^2,<br>Non smokers<br><br>Duration: 4 weeks (3 days/week)	pain complaints at least once per week,<br>iii)body mass index (BMI) < 30 kg/m2 and<br>iv) non-smoker patients (current or past).<br>**Exclusion criteria:**<br>i) pain in any other non-related body area,<br>ii) history of neck pain of traumatic origin,<br>iii) clinical abnormalities or surgeries of the thoracic cage or vertebral column,<br>iv)occupational industrial exposures,<br>v) serious comorbidities and<br>vi) malignancies	**Control group (n=20):**<br>Traditional physical therapy program only	**Deep cervical muscle strength:** Craniocervical Flexion test<br>**Spirometry assessed:**<br>MVV, PEF, FVC, FEV1<br><br>All outcomes evaluated at baseline and after treatment<br><br>*A priori sample size calculation performed to detect a between-group significant difference (a=0.05), power of 95% and large effect size (d=0.8).*	rate in the study group only (p = 0.0001). There was a statistically significant improvement in NDI (p = 0.0001), VAS (p= 0.0001), FVC (p = 0.002) and FEV1 (p= 0.01) in both groups, however, there was no statistically significant difference between both groups.
**Thongtipmak et al 2019 [35]**<br><br>**Aim:** to evaluated the immediate effects and acceptability of a smartphone application named NeckProtector to promote neck pain self-management through stretching incorporating deep slow breathing,	Randomized controlled trial,<br><br>Participants: 100 (Female:79, Male:21),<br>**Treatment group:**<br>mean age: 22.86 ± 1.99 years,<br>mean body mass index: 21.43 ± 3.24 kg/m2<br>**Control group**:<br>mean age 22.68 ± 2.23 years,	**Inclusion criteria:**<br>i) aged between 18 and 34 years<br>ii) had at least 1-year smartphone usage experience, used a smartphone for at least 4 hours per day<br>iii) experienced pain intensity between 3 to 6 (mild to moderate) on the (VAS) during past 3 months<br>iv) total score of the NDI below 40%<br><br>**Exclusion criteria:** patients who:<br>i) had a history of neck or shoulder surgery, including any condition	**Intervention group (n=50):**<br>exercise program via smartphone application<br><br>**Control group (n=50)**:<br>rested for 20 minutes<br><br>all participants for acceptability testing by navigation of the application for 20–30 minutes	**Pain intensity**: VAS,<br>**Muscle tension:**<br>**Function**: CROM,<br>**Pressure pain threshold:**<br>**Acceptability assessment:** System Usability Scale (SUS)<br><br>All outcomes evaluated at baseline and after intervention<br><br>*A priori sample size calculation performed to detect a between-group significant difference*	The immediate effects showed statistically significant improvements in pain intensity, muscle tension, PPT, and CROM in the treatment group in comparison to the control group.<br>Participants reported a high level of acceptability through ratings on a Likert scale. The qualitative questionnaires received

among smartphone users with neck pain.	mean body mass index: 22.33 ± 3.21 kg/m², <br>Duration: 1 session 15–20' min	contraindicating stretching exercise; ii) had any other treatment for neck pain within the past month, such as manipulation, ultrasound diathermy, medication. iii) had reported an underlying disease or disorder that would limit their ability to perform the proposed exercise (e.g., rheumatoid arthritis, degenerative disc diseases, shoulder diseases, heart disease, asthma, and neurological deficits)		*(a=0.05), power of 80% and medium effect size (d=0.5).*	positive responses that the application was easy to use and the exercise program was useful to cope with neck pain
**López-de-Uralde-Villanueva et al. 2020 [21]** <br>**Aim:** To determine the effectiveness of a therapeutic patient education (TPE) intervention based on a biobehavioral approach combined with manual therapy (MT) to reduce pain in patients with chronic nonspecific neck pain.	Randomized controlled trial, <br>Participants: 47 (Female/Male: 36/11), Age: 18-65 years, BMI: 23.5 ± 4.85 kg/m² <br>Duration: 4 weeks (40'/day 2 days/ week)	**Inclusion criteria:** i) 18–65 years of age ii) reported neck pain for at least 12 weeks (chronic pain iii) able to understand, write, and speak Spanish; and iv) a willingness to undergo treatment for pain localized in the neck region. v) a minimum pain intensity of 20-mm in VAS <br>**Exclusion criteria:** i) medical red flags (eg any type of cancer, fracture, metabolic diseases, rheumatologic disease, and osteoporosis);	**Control group (n=20):** Manual Therapy according to Maitland concept plus thoracic spine thrust manipulation <br>**Intervention group A (n=20):** Manual Therapy (MT) plus therapeutic patient education (TPE) (including diaphragmatic breathing) based on a behavioral approach <br>**Intervention group B (n=19):** Manual Therapy (MT) plus therapeutic patient education (TPE)	**Pain intensity:** VAS, **Pain catastrophizing:** Pain Catastrophizing Scale, **Symptom severity and global improvement**: Clinical Global Impression Scale (CGIS), **Mechanosensitive of the median nerve:** Upper Limb Neural Test (ULNT) dual digital inclinometer **Cervical region:** Modified Passive Neck Flexion Test (MPNFT) dual digital inclinometer <br>All outcomes evaluated at baseline, at end of treatment	Statistically significant differences in pain intensity were found when B group was compared with A group and the control group at 4 months (p = 0.015 and p = 0.001, respectively), but no difference was found between A and the control group at the same follow-up period (p = 0.86). Int showed statistically significant differences in all of the secondary outcomes except for pain catastrophizing when

		ii) a history of cervical surgery or whiplash trauma,<br>iii) neck pain with cervical radiculopathy; myelopathy;<br>iv) neck pain accompanied by vertigo caused by vertebrobasilar insufficiency or accompanied by non-cervicogenic headaches<br>vi) fibromyalgia syndrome<br>vii) if they had received some type of pain treatment, including medication and physical therapy, during the previous 3 months, or<br>viii) had pending legal action (e.g., compensation for injury and/or labor), psychiatric disorders, or other<br>problems that could contraindicate the use of the techniques in this study.<br>ix) patients who took any type of medication and/or received other manual therapies during the treatment or follow-up period	based on a behavioral approach plus therapeutic exercise (TE) and neural self-mobilization.<br><br>The patients were asked to perform the exercises at least once per day at home from post-treatment to the 4-month follow-up	(4 wks) and at 16 wks follow up period<br><br>*A priori sample size calculation performed for VAS (primary outcome) to detect a between-group moderate effect size (d=0.25), significance level (a=0.05) and power of 90%.*	compared with the control group at 4 months
**Sakuna et al. 2020 [25]**<br><br>**Aim** to evaluate the effects of breathing retraining with chest wall mobilization on the onset of accessory breathing muscle recruitment and	Randomized controlled trial,<br><br>Participants 32, (Female25/ Male7),<br>Age: 18-45 years,<br>BMI: 18.5–24.9 kg/m^2,<br>Duration: 1 session for 30ˈmin	**Inclusion criteria:**<br>i) at least a 6 monthσ history of neck pain, mild to moderate pain intensity (VAS scale 2–7),<br>ii) body mass index (BMI) 18.5–24.9 kg/m^2, and<br>iii) age between 18 and 45 years old.<br>**Exclusion criteria:**<br>i) severe pain (VAS scale > 7),	**Intervention group (n=16)**:<br>Breathing retraining with chest wall mobilization<br><br>**Control group (n=16)**:<br>rest for 30 minutes	**Muscle activity:** EMG for sternocleidomastoid (SCM), anterior scalene (SC), and upper trapezius (UT) muscles bilaterally<br>**Respiratory parameters:** MVV with Spirometer Spirolab III,<br>**Chest expansion:** flat measuring tape	Significant improvement in respiratory reserve was observed in the intervention group compared to control group through prolonged EMG onset of accessory breathing muscles.

respiratory reserve in patients with chronic neck pain.		ii) any neurological symptoms or iii) known contraindications		**Pain Intensity**: VAS<br><br>All outcomes evaluated at baseline and at the end of treatment<br><br>*A priori sample size calculation performed to detect a between-group significant difference (a=0.05), power of 80% and medium effect size (d=0.5).*	Moreover, increase of MVV, chest expansion** and decrease in pain intensity*** were observed.<br><br>Significant increase in the MVV in the intervention group (91.14 to 94.96 l/min, p =0.041) On the contrary, a significant decrease in chest expansion in the control group (no statistically significant differences of MVV between-groups).<br>Significant differences of chest expansion was found (p = 0.009) between the two groups.<br>Significant differences of pain at rest and pain on worst movement (p = 0.048<br>and p = 0.002, respectively) between groups
**Akodu et al. 2020 [31]**<br><br>**Aim:**<br>To evaluate the efficacy of neck	Randomized controlled trial,<br><br>Participantsς: 14 (Male: 6,	**Inclusion criteria**:<br>i) neck pain lasting for at least 12 weeks	**Intervention group A (n=4)**:<br>neck stabilization exercises,	**Pain Disability Index**: NPDI,<br>**Insomnia severity Index:** ISI<br>**Anxiety and Depression**: HADS,	Neck stabilization exercise combined with isometric exercise improved pain-related disability (p=0.04),

stabilization and isometric neck exercises on pain-related disability, sleep disturbance, psychological status and cardiopulmonary parameters in patients with non-specific chronic neck pain (NSCNP)	Female: 8)<br>Age: 22-62 years<br>BMI: 24.90 ± 3.87 kg/m2<br><br>Duration: 3 sessions over a 4-week period lasting	**Exclusion criteria:**<br>i) cognitive limitations or<br>ii) history of cardiovascular, pulmonary or endocrine disease,<br>iii) health conditions which might prevent them from participating in exercise,<br>iv) chronic neck pain with symptoms indicating a particular red flag	**Intervention group B (n=5):**<br>neck stabilization exercises plus isometric neck exercises,<br><br>**Control group (n=5)**<br>isometric neck exercises only	**Cardiovascular Parameters:** Heart Rate (HR), Blood Pressure (BP), Rate Pressure Product (RPP),<br>**Pulmonary parameters:** Respiratory rate (RR), Borg scale, FVC, FEV1, PEFR<br><br>All outcomes evaluated at baseline, and after 4 weeks intervention<br><br>*A priori sample size calculation performed for Pain (primary outcome) to detect a between-group significant difference (a=0.05) and power of 84%.*	anxiety (p=0.04) and depression (p=0.04) after 4 weeks intervention. Some pulmonary parameters, FVC (p=0.05, p=0.02), FEV1 (p=0.02, p=0.01) improved significantly post intervention in both neck stabilization exercise alone and neck stabilization combined with isometric neck exercise groups. While PEFR (p=0.02, p=0.01) improved significantly in both neck stabilization combined with isometric neck exercises and isometric neck exercise group alone post intervention.
**Simoni et al. 2021 [20]**<br><br>**Aim:** To investigate the effects of	Randomized controlled trial,<br><br>Participantsς: 40 (Male: 10 Female: 30)	**Inclusion criteria**:<br>i) aged between 18 and 65 years<br>ii) neck pain lasting for at least 3 months, without gender distinction	**Intervention group (n=20)**:<br>Standard cervical physiotherapy (including cervical spine manual	**Pain**: NPRS,<br>**Disability**: NDI,<br>**Function:** CROM: Bubble Inclinometer, Pressure Pain	Combined permutation test showed a significant higher improvement in DMT group compared to SDT

diaphragm manual therapy associated with<br>standard physiotherapy treatment on pain in patients with Chronic Neck Pain (CNP).	Age: 22-62 years<br>BMI:<br>DMT: 22.64±3,14<br>SDT: 21.98 ± 3.77 kg/m2<br><br>Duration: 3 sessions over a 4-week period lasting 30 min each	**Exclusion criteria:**<br>i) contraindications of cervical treatment physiotherapy or osteopathic treatments in the previous 3 months,<br>ii) rheumatic, oncological or respiratory pathologies,<br>iii) spinal, thoracic or abdominal surgery in the previous 3 years,<br>iv) whiplash,<br>v) previous cervical fractures,<br>vi) cervical anatomic anomalies (deformities),<br>vii) thrombotic events; > 37° body temperature in the 48 h before treatment,<br>viii) Body Mass Index (BMI) > 30,<br>ix) pregnancy	therapy & soft tissue treatment) and Diaphragm Manual Therapy (DMT) consisting of "Manual Diaphragm Release Technique" and "Doming the Diaphragm"<br><br>**Control group (n=20):** Standard cervical physiotherapy and sham Diaphragm Manual Therapy (DMT)	Threshold (PPT): digital pressure algometer<br>**Quality of life**: SF-36,<br>**Adverse events:** questionnaire<br><br>All outcomes evaluated at baseline, after each session, at 3 and 6-months<br><br>*A priori sample size calculation performed for Pain (primary outcome) to detect a between-group significant difference (a=0.05) and power of 90%.*	group (p-value = 0.0002).<br>The between-group comparisons on single outcomes showed a statistically significant improvement only for pain pressure threshold on upper trapezius (adjusted p-value = 0.029).<br>No adverse events related to the intervention were registered.

**Dareh-deh et al 2021 [22]**<br><br>**Aim:** to compare the effect of a therapeutic program with and without respiratory exercises on smartphone users with FHP and NSCNP	Randomized controlled trial,<br><br>Participants: 60 (Male: - Female:)<br>Age: 22-27 years<br>BMI: 21-25 kg/m2<br><br>Duration: 3 sessions over an 8-week period lasting 45 to 60′ min per session	**Inclusion criteria:**<br>i) males and females who were using a smartphone for more than four hours a day<br>ii) who<br>rated their worst pain over the last 24-h as moderate using the visual analogue scale (VAS),<br>iii) with neck disability<br>index (NDI) scores between 28 and 45%, and<br>iv) pain lasting longer than three months<br>v) FHP was defined as a<br>cervical angle < 5<br><br>**Exclusion criteria:**<br>i) previous history of neck or back surgery,<br>ii) neurological signs,<br>iii) rheumatoid arthritis and<br>iv) currently using muscle relaxation medication.	**Intervention group A (n=20):**<br>Combined respiratory and therapeutic<br>exercise<br><br>**Intervention group B (n=20):**<br>Therapeutic exercises alone<br><br>**Control group (n=20):**<br>received a pamphlet including information on postural corrections<br>and improving general health	**Pain:** VAS,<br>**Forward Head Posture:** photogrammetry,<br>**Activity of specific muscle:** EMG<br>**Respiratory pattern:** MARM<br><br>All outcomes evaluated at baseline, and after 8 weeks intervention<br><br>*A priori sample size calculation performed for Pain (primary outcome) to detect a between-group significant difference (a=0.05) and power of 90%.*	Respiratory exercises plus a therapeutic program had no extra benefit on pain intensity,<br>forward head angle and muscle activity compared to the same therapeutic<br>program without respiratory exercises. Furthermore, despite respiratory pattern, none of the secondary outcomes<br>proved superior in the combination group.

**Arif et al 2022 [26]**<br><br>**Aim:** to determine the effects of cervical stabilization exercises on respiratory strength in chronic neck patients with forward head posture.	Randomized clinical trial,<br><br>Participants: 40 (Female:28, Male:12), Age: 18-55years, BMI: <30 kg/m2<br><br>Duration: 4 weeks (3 days/week)	**Inclusion criteria:**<br>i) aged between 18 and 55 years,<br>ii) with NDI-Urdu score (5-15) mild neck disability,<br>iii) forward head posture with craniovertebral angle (CVA<50°),<br>iv) cervical pain for at least three months<br><br>**Exclusion criteria:**<br>i) traumatic neck pain,<br>ii) thoracic or vertebral column surgery,<br>iii) BMI of more than 30,<br>iv) smoking history,<br>v) any red flag signs	**Intervention group (n=20):** Cervical stabilization exercises plus Conventional physiotherapy**,**<br><br>**Control group (n=20):** Conventional physiotherapy: Heating pad, TENS, Cervical isometric exercises	**Pain Intensity:** NPRS**,**<br>**Disability:** NDI,<br>**Forward head posture:** CVA,<br>**Respiratory Strength:** SINGLE BREATH COUNT,<br>**Respiratory Function:** 'SPIROMETRY' (outcome not specified)<br><br>All outcomes evaluated at baseline and at 4th week of treatment<br><br>*A priori sample size calculation performed to detect a between-group significant difference (a=0.05), power of 95% and large effect size (d=0.8).*	Post- intervention results were significant differences in both groups (p<0.05) with the intervention group showing more improvement.

**Anwar et al 2022 [27]**<br><br>**Aim:** to evaluate the effectiveness of breathing re-education plus routine physical therapy in patients with NSCNP	Randomized clinical trial,<br><br>Participants: 14 (Female:8 / Male:6), Age: 25-50 years, BMI: 25,37±8,26 kg/m2<br><br>Duration: 8 weeks (3 days/week)	**Inclusion criteria:**<br>i) having neck pain for at least 3 months,<br>ii) being 25 to 50 years of age,<br>iii) no history of respiratory disease and<br>iv) FEV1/FVC ratio not less than 60%.<br><br>**Exclusion criteria:**<br>i) upper cervical symptoms, such as dizziness, dysphagia or dysarthria,<br>ii) post-traumatic neck pain,<br>iii) allergic asthma,<br>iv) depression	**Intervention group A (n=30):**<br>Routine physical therapy treatment with infrared radiation (IRR) plus isometric exercises for the muscles of cervical spine<br><br>**Control group (n=28):**<br>the same Routine physical therapy plus supervised breathing exercises<br><br>A session lasted about 20-25' min	**Pain intensity:** VAS,<br>**Range of Motion (ROM):** CROM Basic devise USA,<br>**Disability:** NDI,<br>**Cervical muscle strength and endurance:** handheld neck dynamometer and bio-pressure feedback,<br>**Spirometry:** FEV1, FVC, FEV1/FVC<br><br>All outcomes evaluated at baseline on 4th and 8th week of treatment<br><br>*A priori sample size calculation performed to detect a between-group significant difference (a=0.05), power of 95% and large effect size (d=0.8).*	There were significant improvements in all outcomes for breathing re-education group for all cervical measurements at 4th and 8th week. Also, there were more significant improvements in between groups for breathing re-education group.

Abbreviations: **ADIQ:** Athletes Disability Index Questionnaire, **BHT**: Breathing holding time, **BP:** Blood Pressure, **CGIS:** Clinical Global Impression Scale, **CVA:** Craniovertebral Angle, **CWE:** Chest wall expansion, **DASS21**: Depression, Anxiety, Stress Scale 21, **ELHQ:** Everyday Life-Lifestyle-Health Questionnaire, **EMG:** electromyographic, **$ETCO_2$:** End Tidal CO2, **FAB-Q:** Fear-Avoidance Belief Questionnaire, **FEV1:** Forced Expiratory Volume in 1 second, **FVC:** Forced Vital Capacity, **FHP:** forward head posture, **HADS:** Hospital Anxiety and Depression Scale, **HLC:** Health Locus of Control Scale, **HR:** Heart Rate, **K-ODI: (**Korean) Oswestry Disability Index, **LOS**: Limits of Stability, **MIP:** maximum inspiratory pressure, **MEP:** maximum expiratory pressure, **mFRT:** modified Functional Reach Test, **mLRT:** modified Lateral Reach Test, **MODQ:** Modified Oswestry Disability Questionnaire, **MPNFT**: Modified Passive Neck Flexion Test, **MVV:** Maximum Voluntary Ventilation, **NDI:** Neck Disability Index, **NFME:** Neck Flexor Muscle Endurance Test, **NPRS:** numerical pain rating scale, **NRS:** numeric rating scale, **NSCNP:** non-specific chronic neck pain, **NQ:** Nijmegen Questionnaire, **PCS:** Pain Catastrophizing Scale, **PHQ4:** Patient Health Questionnaire, **PEF:** Peak Expiratory Rate, **PIF:** Peak Inspiratory Flow, **PPT:** Pressure Pain Threshold, **PSS:** Perceived Stress Scale, **PSFS:** Patient Specific Functional Scale, **RPP:** Rate Pressure Product, **RR**: Respiratory Rate, **QVAS:** Quadruple Visual Analogue Scale, **RMQ:** Roland-Morris Disability Questionnaire, **RR:** Respiratory Rate, **SF-36:** Short Form Health Survey, **SF-MPQ:** Short-form McGill Pain Questionnaire, **SMIP**: Sustained Maximal Inspiratory Pressure, **SOT:** sensory organization test, **SUS:** System Usability Scale, **TFBS:** Total Faulty Breathing Scale, **TSK:** Tampa Scale of Kinesiophobia, **TSS:** Treatment Satisfaction Scale, **ULNT:** Upper Limb Neural Test, **VAS:** visual analogue scale, **VAFS:** Visual Analog Fatigue Scale, **VC:** Vital Capacity, **wk:** week.
